# AAT4IRS: automated acceptance testing for industrial robotic systems

**DOI:** 10.3389/frobt.2024.1346580

**Published:** 2024-10-03

**Authors:** Marcela G. dos Santos, Sylvain Hallé, Fabio Petrillo, Yann-Gaël Guéhéneuc

**Affiliations:** ^1^ Départment d’Informatique et Mathématique, Université du Québec à Chicoutimi, Chicoutimi, QC, Canada; ^2^ Département de génie logiciel et TI, École de Technologie Supérieure (ÉTS), Montréal, QC, Canada; ^3^ Department of Computer Science and Software Engineering, Concordia University, Montréal, QC, Canada

**Keywords:** robotics, industrial robots, software testing, automated testing, acceptance testing

## Abstract

Industrial robotic systems (IRS) consist of industrial robots that automate industrial processes. They accurately perform repetitive tasks, replacing or assisting with dangerous jobs like assembly in the automotive and chemical industries. Failures in these systems can be catastrophic, so it is important to ensure their quality and safety before using them. One way to do this is by applying a software testing process to find faults before they become failures. However, software testing in industrial robotic systems has some challenges. These include differences in perspectives on software testing from people with diverse backgrounds, coordinating and collaborating with diverse teams, and performing software testing within the complex integration inherent in industrial environments. In traditional systems, a well-known development process uses simple, structured sentences in English to facilitate communication between project team members and business stakeholders. This process is called behavior-driven development (BDD), and one of its pillars is the use of templates to write user stories, scenarios, and automated acceptance tests. We propose a software testing (ST) approach called automated acceptance testing for industrial robotic systems (AAT4IRS) that uses natural language to write the features and scenarios to be tested. We evaluated our ST approach through a proof-of-concept, performing a pick-and-place process and applying mutation testing to measure its effectiveness. The results show that the test suites implemented using AAT4IRS were highly effective, with 79% of the generated mutants detected, thus instilling confidence in the robustness of our approach.

## 1 Introduction

According to the International Federation of Robotics, the operational stock of industrial robotic systems (IRS) is increasing. In 2022, the number of robot installations hit a record high, reaching 553,052 units. This marked the second consecutive year with over 500,000 units, reflecting a 5% increase from the previous year and a compound annual growth rate (CAGR) of 7% from 2017 to 2022. The operational stock of industrial robots also experienced significant growth, reaching 3,903,633 units—an increase of 12%—with an average annual growth of 13% over the past 5 years. Despite a slowing global economy, the forecast for 2023 predicts over 590,000 global robot installations (IFR, 2024). The increase in the number of industrial robotic systems operating in the most diverse environments also increases the necessity for these systems to handle failures and meet quality aspects.

Conventional software systems can be defined as “failing” when customers’ expectations have not been met and/or when the software does not help the customer (Chillarege, 1996). To prevent this, developers and stakeholders must use automated software testing to improve fault detection in the system, which will be reflected in improved software quality (Naik and Tripathy, 2018).

Our aim is to improve fault detection in IRS by applying a specific software testing approach. Some research has applied software testing in robotic systems (Bretl and Lall, 2008; Estivill-Castro et al., 2018; Mossige et al., 2015; Chung and Hwang, 2007; Erich et al., 2019; Nguyen et al., 2023). In addition, there are some systematic literature reviews and surveys on related topics that we used to clarify the issues and challenge to apply software testing to robotic systems (Afzal et al., 2020; Afzal et al., 2021).

Afzal et al. (2020) conducted a qualitative study on the challenges of testing robotics systems. They identified five testing challenges to writing and designing for robotic systems: unpredictable corner cases, engineering complexity, culture of testing and coordination, collaboration, and documentation. The coordination, collaboration, and documentation challenge according to the authors is “…the lack of proper channels for coordination and collaboration among multiple teams and a lack of documentation.”

According to Afzal et al. (2020), one of the significant challenges in **coordination, collaboration, and documentation** stems from the need for adequate channels for coordination and collaboration among multiple teams and for more documentation. Coordination within many robotics companies often requires bridging gaps between teams with diverse backgrounds, such as software and hardware teams. Additionally, it is common to encounter the need to integrate and write tests for third-party components without any accompanying documentation. More standards and guidelines for writing and designing tests for robotic systems must also be developed.

Another challenge identified based on participants’ responses concerns the **culture of testing**. One defining feature of the robotics community is its ability to bring together individuals from various disciplines, such as electrical and mechanical engineering. This diversity not only drives significant advances in robotics but also poses certain challenges. For example, a representative quote from one of the respondents is, “The world of robotics unites folks from different backgrounds. Folks from a software background might observe testing differently from those who are not.”

Behavior-driven development (BDD) is a software development approach that promotes collaboration between technical and non-technical stakeholders during the development process. It introduces a common language made up of structured sentences expressed in natural language. This language aims to improve communication and understanding between project team members and business stakeholders, resulting in more effective software development and clearer alignment with business objectives (Irshad et al., 2021).

Solis Pineda and Wang (2011) identified six key characteristics of BDD: (i) the use of ubiquitous language based on business terminology; (ii) an iterative decomposition process for high-level specifications; (iii) templates to write user stories and scenarios; (iv) automated acceptance tests; (v) readable specification code; (vi) elaboration of behaviors based on the needs of the development phase. The software testing applied in BDD is automated acceptance testing that emphasizes the validation of a system’s performance within the context for which it is intended.

Our objective is to streamline the software testing process for IRS through the introduction of a specialized approach we have developed known as AAT4IRS (Automated Acceptance Testing for Industrial Robotic Systems). This ST approach entails customizing and implementing automated acceptance templates derived from the principles of BDD. By leveraging this approach, we aim to enhance IRS fault detection.

To evaluate our methodology in an industrial setting, we developed a test suite to apply our approach. The scenario involved an industrial robot picking items and placing them into a designated box based on color. Pick-and-place behavior is utilized in almost all industrial environments that use industrial robotics to automate processes. This scenario is similar to that (and the requirements) used in robotics competitions and benchmarks. Our decision to use as our inspiration the requirement used in a robotics competition was because of the absence of public repositories of more real requirements (used in industry) available (Nguyen et al., 2023).

The effectiveness of our approach was evaluated through mutation testing. The mutation score serves as a reliable metric for assessing the efficacy of a test set in identifying faults. Research indicates that achieving a higher mutation score markedly improves the detection of faults (Jia and Harman, 2011; Papadakis et al., 2018). As robotic systems interact with the real world, we created “mutants” to simulate these interactions. Thus, we implemented mutation testing to evaluate our test suite in the context of robotic systems.

The initial results show that the test suite implemented using AAT4IRS was able to kill 79% of the mutants. These results show the effectiveness of a test suite implemented by following AAT4IRS. Despite the benefits observed in the proposed approach, it is still open to improvement. We detail such future possibilities in Section 8.

The main contributions of this study are (i) a software testing approach to apply automated acceptance testing in IRS (Section 3), (ii) an empirical study to evaluate our approach (Section 4), and (iii) an initial guideline to create mutants for industrial robotic systems (Section 5).

The remainder of this paper is organized as follows: Section 2 provides background on the core concepts for our study (industrial robotics systems, acceptance and mutation testing) and related work. Section 6 discusses our results and highlights the observed benefits. Section 7 discusses the threats to validity. Section 8 synthesizes final remarks and future work.

This paper is an extended version of a demo paper (Santos et al., 2022), with the following changes: (i) expanded explanations for the proposal approach; (ii) mutation testing to evaluate it; (iii) initial guideline to create mutants for IRS.

## 2 Background and related work

### 2.1 Industrial robotic systems

An “industrial robotic system” (IRS) is a system composed of industrial robots, end-effectors (grippers, magnets, vacuum heads), sensors (visual, torque, collision detection, 3D vision), and equipment (belt conveyors). As with any robot in general, an industrial robot is a complex system comprising both hardware and software; as such, it can be subject to failure in any of these components. The scope of our study is on the software component, which is composed of two parts. First, the *control layer* is responsible for translating commands so that the IRS can understand and execute them; thus, it is essentially a collection of drivers interacting with the hardware. Second, the *application layer* is composed of a software script which defines the robot’s desired behavior according to business requirements (Ahmad and Babar, 2016; ISO/IEC 8373, 2012).

According to Heimann and Guhl (2020), the methods of programming industrial robots can be classified based on the interaction between the operator (who is responsible for programming and operating the industrial robot) and the robot. These methods can be online, offline, or hybrid. In the first *online* method, the operator programs the robot on the shop floor, using either the “lead through” method (the operator takes the robot manually and guides it through a trajectory) or the “teach-pendant” method (the operator guides the manipulator to specific points, records these points to compose a trajectory, and the manipulator executes the trajectory). The shop floor must stop its production from having the IRS programmed in both.

In contrast, in the *off-line* mode the operator uses an environment composed of industrial robot programming languages (IRPLs) and/or simulation software. IRPLs are purpose-built, domain-specific programming languages that include special instructions to move the robot’s arm(s), as well as standard control-flow instructions and APIs to access low-level resources (Pogliani et al., 2020).

Finally, in the *hybrid* method, the operator works offline to create the flow and calibrate and validate the physical system (online). The hybrid method thus utilizes the benefits of online and offline methods.

Validation in IRS depends on the method used. When the online method is applied, the operator must stop the shop floor, program the robot, and make it execute the program. The robot is then evaluated to determine whether it had the expected behavior by visual checking and/or with a reading sensor (if some are available in the environment).

In the offline method, the validation process takes place in a simulator; only if the robot behavior fits the business requirement (BR) can the program (i.e., the application layer) be sent to the robot on the shop floor. The validation process uses the operator’s knowledge and experience with the expected robot behavior.

In the hybrid method, the validation also used the simulators, after the operator fine-tunes the program using the online method to make real-time adjustments based on the robot’s behavior interacting with the physical environment.

In summary, although these are different methods of programming IRS, they have the same aspect concerning validation, which uses the operator’s knowledge and expertise about the expected robot behavior. Furthermore, the validation process is manual for each program written. Suppose that there are changes to the BR (modification or addition of a feature). In that case, the operator must change and validate the program again, even if they validated part of the program before the modification. This manual aspect of code validation in an industrial environment increases project time and cost; automated software testing is thus needed for industrial robots.

### 2.2 Automated acceptance testing (AAT)

There are various levels of software testing, one of which is the *acceptance test*. This type of testing aims to check if the system meets a set of acceptance criteria (AC) that guarantee that its quality is suitable for the particular business requirements.

As with other forms of testing, acceptance testing can be applied manually or using automation tools. The benefits of test automation are increased test productivity, better coverage of regression tests, reduced duration of testing phases, reduced cost of software maintenance, and increased effectiveness of test cases. With test automation, an organization can create a rich library of reusable test cases, facilitating the execution of a consistent set of test cases (Naik and Tripathy, 2018).

However, introducing automation creates a gap between the business requirements and technical aspects of software testing. On the one hand, business teams write the BRs and use them in the definition of AC; on the other hand, these requirements must result in the generation and execution of test cases.

To align the business and technical needs of software, behavior-driven development (BDD) can be a good choice (Irshad et al., 2021). In BDD, one typically uses a high-level human-readable language, such as Gherkin (Wynne and Hellesoy, 2012), to bridge the gap between BRs and technical aspects. On the one hand, the document describing the requirements is easily readable by developers, QA teams and business teams. On the other hand, the development team uses the same document to automate the execution of acceptance tests (Nicieja, 2017).

In BDD, the AC for each business requirement is described in two parts: the *feature* and the *scenario*. The feature is a deliverable piece of functionality to allow the business to achieve its goals. It is described using the user story format: “**As a** [description of the user], **I want** [functionality] **so that** [benefit].” The template to describe scenarios in BDD is: “**Given** [pre condition for the scenario and test environment], **when** [action under test], **then** [expected outcomes]” (Smart, 2014). A scenario is itself composed of *step definitions* responsible for interacting directly with the system.

### 2.3 Mutation testing

Mutation testing is a software testing technique in which the original code suffers some changes called “mutated versions” or “mutants”. These mutants represent different potential defects that are modifications in the source code. These modifications include changing a mathematical operator, swapping the order of two statements, or replacing a conditional with the opposite. The mutation score is the percentage of killed mutants (detected by the test suite) with the total number of mutants.

In order to apply mutation testing, we used the test suite against each mutant. If the tests detect the changes made to create the mutants, the mutants die. If the test suite does not detect the error, the mutants survive. The number of mutants killed and that survive is used as a metric to evaluate the effectiveness of the test suite (Jia and Harman, 2011).

According to Jia and Harman (2011), mutation testing can measure the effectiveness of a test suite in terms of its ability to detect faults. ISO/IEC 25010 defines “effectiveness” as the “…accuracy and completeness with which users achieve specified goals” (ISO/IEC 25010 2011).

Our decision to use a mutation-based approach for evaluating the testing campaign is based on its ability to replicate a wide range of scenarios, surpassing the limitations of variations in environmental descriptions found within scenario files from BDD alone. Our methodology intentionally integrates non-deterministic mutants to emulate unpredictable behaviors commonly encountered in simulation and robotic systems, such as sensor noise and fluctuations in simulation speed.

Additionally, we drew inspiration from previous studies where mutation testing has been successfully applied to assess cyber-physical systems. For instance, Leotta et al. (2018) conducted a thorough investigation into automated acceptance testing for IoT systems, including sensors/actuators, smartphones, and remote cloud-based infrastructure. They evaluated their approach using mutation testing and demonstrated its practical application. Similarly, Afzal (2021) presented an innovative automated testing framework using software-in-the-loop simulation for cyber-physical systems. Their use of mutation testing showcased the effectiveness of their approach in evaluating system performance and robustness.

### 2.4 Related research

We consider related research for our study, a primary study that performed software testing activity (design and generate test cases) at different levels (unit, integration, acceptance, system) for a type of robotic system (mobile, industrial).

In this study, Nguyen et al. (2023) analyzed robotic application requirements and acceptance criteria, explicitly focusing on robotic competitions and benchmarks. They aimed to address the challenges of representing and managing requirements in the context of robotic applications’ increasing complexity and diversity. We consider this research to be complementary to ours. In both studies, the authors applied BDD to rewrite the requirements for IRS. However, Nguyen et al. (2023) highlighted the application of BDD to express and manage requirements for robotic applications, emphasizing the potential for introducing automation into verifying and validating these requirements in robotics. Our study used BDD to rewrite the requirements for industrial robotic systems and implement executable tests using a simulator.

Ashraf et al. (2020) proposed coverage criteria for white-box testing to test industrial robot tasks and a framework to automatically generate the test cases to achieve the coverage criteria defined by them. However, our research does not aim to automatically generate test cases.

Breitenhuber (2020) proposed an application-level testing framework for robot software applications that uses known robotic software to describe the expected behavior of an application or its components. They focus on evaluating the component behavior in robotics systems that use the ROS framework in application-level testing. They apply the testing tools available in the ROS environment to test the components. In our study, we aimed to apply automated acceptance for industrial robotic systems in general, not just in ROS-based systems. Furthermore, in our approach, acceptance testing is automated, and the BR is translated to the BDD template for AC.

Erich et al. (2019) presented a framework for automatically testing applications for collaborative robots and demonstrated the proposal in a case study for automatically testing a pick-and-place application. Their proposal was a framework applied in a physical environment and at the level of integration testing. Ours focuses on acceptance testing, leading us to consider their research and ours to be complementary.

Estivill-Castro et al. (2018) proposed a robot simulator following the model-view-controller software pattern. They use simulators with the stripped GUI under a continuous integration paradigm for robots to scale up the testing integration with robot behavior. The simulator was an environment to be applied in our approach and was not developed in our study.

Mossige et al. (2015) presented cost-effective automated testing techniques to validate complex industrial robot control systems in an industrial context and employed their methodology in continuous integration and constraint-based testing techniques. Although our research was also focused on industrial robotic systems, we focused on automated acceptance testing, so our studies are complementary.

Alexander et al. (2015) proposed the concept of situation coverage. They empirically evaluated situation coverage by testing a simple simulated autonomous road vehicle and comparing its effectiveness with random test generation. We highlight that the challenges in testing autonomous robots, as summarized by them, are similar to those for testing industrial robots, making our study and theirs complementary. However, they proposed the concept of situation coverage, which measures the proportion of all possible situations tested by a given test set as a potential solution. For them, situations are starting states and rules for projecting future states; they do not commit to a linear sequence of events. Our approach uses features and scenarios written in natural language to guide the test generation. The scenario coverage approach aims to cover a representative set of scenarios described by linear sequences. Therefore, the goal of both approaches is the same (improving software in robotics through software testing), but they use different methods.

Chung and Hwang (2007) presented a testing process and evaluation elements to test the software of intelligent robots. They proposed a test case design methodology based on user requirements and ISO standards for software testing. However, they did not perform acceptance testing using the BRs as input. Our study aims to apply AAT using acceptance criteria defined by BRs.

To our knowledge, our work is the first to apply automated acceptance testing for industrial robotic systems using the BDD template to reduce the effort in discovering faults and to ensure that the application meets specific BRs.

## 3 Proposed approach

Our approach is to apply automated acceptance testing (AAT) to evaluate whether the system meets the business requirement (BR). The system under test (SUT) is the IRS performing the expected behavior defined by the BR. We concentrate on the off-line method, which makes use of simulation.

When testing software for IRS, it is important to consider their unique features and needs. To ensure that the software meets the specific requirements and expectations of the IRS, it is crucial to involve domain experts, stakeholders, and end-users in the testing process.

According to Afzal et al. (2020), robotic systems differ from conventional software in several critical dimensions. Robots are complex systems composed of software and hardware. The latter interacts with the physical world through sensors and actuators, which can lead to errors that are challenging to predict. Furthermore, the notion of correctness is hard to specify.

**FIGURE 1:**
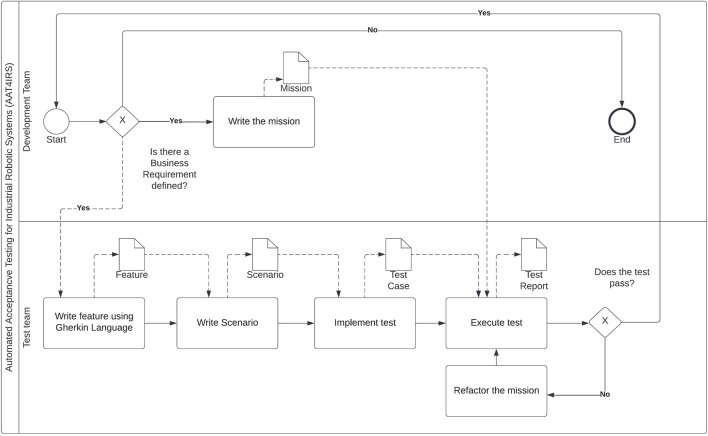
Proposed approach to applying automated acceptance testing for IRS.

Thus, we propose an approach that considers the differences between conventional and robotic systems by validating whether the software meets the needs defined in the BRs. Figure 1 shows our approach, which outlines the necessary activities and corresponding input/output. Starting the AAT4IRS process requires a decisive definition of BRs and acceptance criteria (AC) to ensure that the features developed provide actual business value. Collaboration with stakeholders, including robot operators, engineers, and other relevant parties, is crucial to clearly define the AC for the robot software.

As for conventional IRS systems, the BRs need to describe the business need or problem that requires a solution. These requirements should be measurable and actionable and include AC to ensure that all stakeholders agree on what the system should accomplish.

In our approach, we can take two paths with the BR defined. One starts with the development team *writing the mission* (the application layer defined in Section 2) —essentially the system under test. The other path starts with the test using the Gherkin language to *write the feature*. The feature in our approach will adapt the template defined in BDD and will follow the following format: “**As an** operator, **I want** [process to be automated] **so that** I can automate the [process] using an IRS.”

The next step in AAT4IRS is *writing scenarios*, and we follow the BDD template (Given–When–Then). The *Given* step involves outlining the initial conditions for industrial processing using robots, including robot setup, environment preparation, and sensor calibration. We connect these procedures with the *And* connector in the BDD template. The *When* step outlines the system under test. Lastly, in the *Then* step, we evaluate whether the system meets the expected behavior using the AC defined in the first activity. For example, we can use a sensor to evaluate the final position of a box.

Following the AAT4IRS approach, we implemented the test, where we must implement a function for each sentence defined in each scenario. For example, we will have a function linked with the sentence *Given* defined in the scenario. When we implement the tests, we create the link between the tests and the system under test. For example, if we have the following sentence “Given that the robot is in the initial position…”, we will need to implement a function to put the robot in the initial position and also to assert whether the robot achieves the expected position.

Finally, we need to *execute the tests*. Running them will trigger the functions that access the application layer. If the test passes, the process starts again with another BR. However, if the test fails, the process refactors the mission (application layer) start until the test passes.

The output of the activity *execute tests* is a test report. We aim to improve some important aspects of the software development process for IRS. By following our approach, the software in industrial robots can undergo thorough acceptance testing to ensure that it meets the requirements and expectations of end users. It is important to involve domain experts and stakeholders throughout the acceptance testing process to ensure that the software aligns with the specific needs of the industrial robot application.

Our proposed process draws similarities with applying automated acceptance testing (AAT) to conventional systems. However, crafting features, scenarios, and test cases requires nuanced adaptation that aligns with the demands and objectives of industrial processes. To achieve this, we incorporate industry-specific language when formulating scenarios using the Given–When–Then structure. For example, the Given statement sets the initial conditions, such as the starting position of a robot. Furthermore, we utilize instrumentation tailored to industrial settings to establish AC. By employing positional sensors across three axes, we validate positions and define AC based on sensor characteristics, ensuring alignment with business objectives. Ultimately, our approach entails integrating domain-specific language to customize automated acceptance testing within the BDD standards framework.

## 4 Applying AAT4IRS to pick-and-place task

The use of industrial robots in pick-and-place scenarios is common in competitions and benchmarking exercises. Our decision to draw inspiration from a robotic competition is rooted in the limited accessibility of real-world industrial requirements, as noted by Nguyen et al. (2023). As such, we looked to the Robotic Grasping and Manipulation Competition’s Task Pool for direction. In particular, we turned to a specific task outlined in their competition framework: “Pick Up and Place Using Tongs” (Sun et al., 2018).

The robot model used was the Gen3 from Kinova Robots (2022), and the end-effector is a gripper, the Robotiq-2f-85 from Robotiq (2022). This model has the follow sensors: torque, position, current, voltage, temperature, accelerometer, and gyroscope. We use the position sensor that gives us the arm position for the three axes (x, y, z).

The experiment took place in the esteemed Robot Operating System (ROS) environment, which is highly regarded in the robotics community for its adaptability and reliability. ROS is widely accepted as an ideal platform for developing robotic software due to its extensive range of libraries, tools, and conventions. Its seamless communication between various components simplifies the development of complex robot software systems. As mentioned by Quigley et al. (2015), a ROS-based system involves numerous concurrent programs that exchange messages, enabling effective collaboration and coordination.

For our experiment, we have an industrial environment with an IRS performing a pick-and-place process (an industrial process in which an industrial robot picks up an object from one location and places it in another).

We implement the experiment within simulated environments, which can lower testing expenses and expand opportunities for test automation. Timperley et al. (2018) argue that many real-world robotics bugs could be replicated and addressed in simulation environments. Moreover, simulations mitigate the risk of damaging equipment (Bossecker et al., 2023), eliminate the necessity for physical prototypes (Roth et al., 2003) and offering a cost-effective means to implement changes (Robert et al., 2020).

Therefore, we integrated a suitable simulator within the ROS environment. Among the available options, Gazebo emerged as the leading choice for robotic simulation due to its ability to replicate diverse robotic platforms equipped with standard sensors like cameras, GPS units, and IMUs. Despite operating independently, Gazebo seamlessly integrates with ROS via the “gazebo_ros” package, enabling bidirectional communication (Quigley et al., 2015; Farley et al., 2022).

**FIGURE 2:**
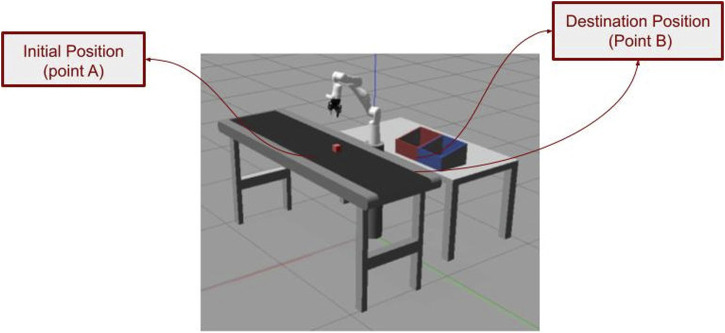
Pick-and-place task performed using a Gazebo simulator.

The robot’s mission performed in our experiment was to transfer a box from its initial position (point A) on the conveyor to its destination at the delivery table (point B) (Figure 2). The robotic arm needed to precisely navigate to the target location and use its gripper to securely grasp the object while ensuring proper alignment. Upon successful pickup, the robotic arm was to carefully place the box on the delivery table before returning to its original position. The instrumentation necessary to run our experiments, besides the IR, is sensors to read the IR, box color, and the box positions.

Following the approach defined at Section 3, we write the *mission* for the IR to meet the follow BR.

BOX 1 **Business requirement.**

We must move the box from the conveyor to the delivery point, respecting the box color. The robot needs to be positioned correctly before the pick-and-place process can begin. The final position of the box should not exceed 0.02 cm in any of the three axes.

We would like to highlight that the AC defined by the BR was related to the box’s final position. However, to perform the pick-and-place process, we also needed the robot’s and box’s initial position. Therefore, we rewrote the BR to add these AC with the acceptable threshold.

BOX 2 **Business requirement.**

We must move the box from the conveyor to the delivery point, respecting the box color. The robot needs to be positioned correctly before the pick-and-place process can begin within a threshold of 0.02 cm in all three axes. For the box, the threshold is 0.01 cm, and also for the three axes. The final position of the box should not exceed 0.02 cm in any of the three axes.

After writing the **mission**, we created the test suite following AAT4IRS. We used the *pytest-bdd* library (Pidsadnyi and Bubenkov, 2022), the most popular framework in Python that implements a subset of the Gherkin language to enable the automating of project requirements testing.

To achieve this, we use Gerkin language to write a **feature**. The first step was to establish the initial environment for our experiment, which involved initializing both the robot and conveyor, moving the robot to the home position, and placing the box in a specific location. Given that each position has three axes, we composed a *Given* statement for each axis, as shown in the Listing 1. The *When* statement involved the pick-and-place automation system we are testing, while the *Then* statement determined whether the box was in the expected position with the maximum error as defined by the AC in the BR.

In order to proceed, we needed to perform a test that created a function for each statement in the scenario. The criteria for accepting are related to the robot and box within specified position limits. The next step was to use the feature to construct the scenario. As the AC were based on data from sensors that monitor the positions of the robot and box, we incorporated sensor readings into the test script to create assertions (Listing 2 and 3). Once the test suite was developed, we ran it against the original code and successfully passed all tests.

## 5 Evaluation

We evaluated AAT4IRS through mutation testing. The mutants defined took into account the fact that the code for a robotic system is not just any piece of procedural code but a specific type of program that manipulates robotic components that interact with the physical world.

**FIGURE 3:**
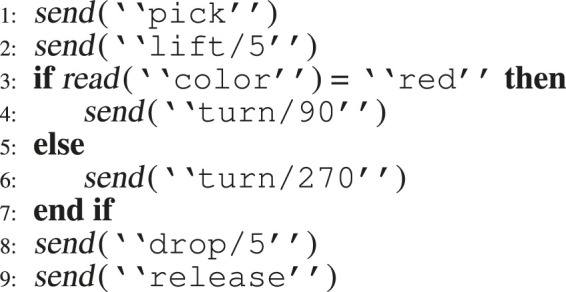
Simple robot program.

Figure 3 shows a pseudo-code for a possible program in which the IR performs the pick-and-place task. The robot picks the box, lifts it from the ground by some amount, and does the reverse operations after turning to two possible angles, depending on the box’s color determined by a visual sensor. The code is mostly a linear sequence of *send* and *read* commands, which, in the context of ROS, give commands to the robot and probe the state of the environment, respectively. The program has few control structures, variables, or arithmetical operations, which are the typical points where mutation operators are applied. There are very few locations where mutations can be injected.

Listing 1 Feature and scenario.

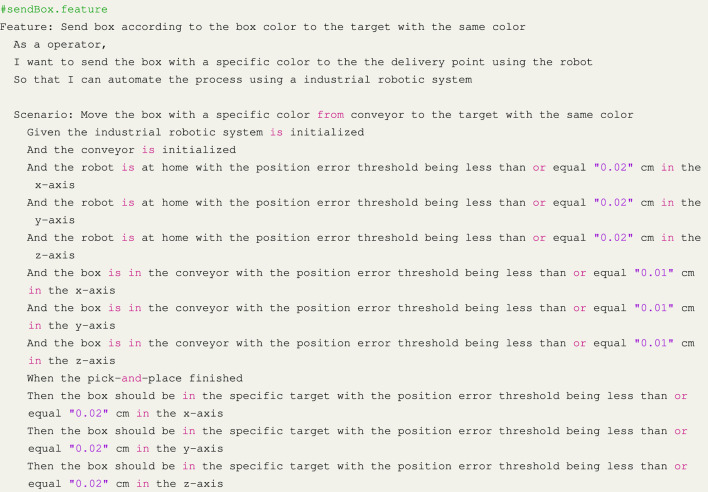

In this context, one could understand that the numbers 90 and −270 mean “left” and “right”, and that confusing one for the other is probably more likely for a developer than providing an angular value that is incorrect by a single degree, such as 90 and −90. We also introduced mutants that replicated errors in the sensor reading. Our goal was to create a comprehensive set of mutants that accurately represent the solution space under examination, and to provide an informative value related to the effectiveness of our approach. Thus, we identified the high-level write/read operations and defined appropriate ways of mutating them. Table 1 shows the possible transitions between the original code and the mutant.

**TABLE 1:** Original X mutant command for robotic systems.

Original	Mutant
rotateleftbyX	rotaterightbyX
rotaterightbyX	rotateleftbyX
translateforwardbyX	translatebackwardsbyX
translatebackwardsbyX	translateforwardbyX
translateforwardbyX	translateforwardtoX
translatebackwardsbyX	translatebackwardstoX
docommandX	donothing
docommandX	docommandXtwice
distancevalueisX	distancevalueis−X(reversedirection)
distancevalueisX	distancevalueisX+noise

**FIGURE 4:**
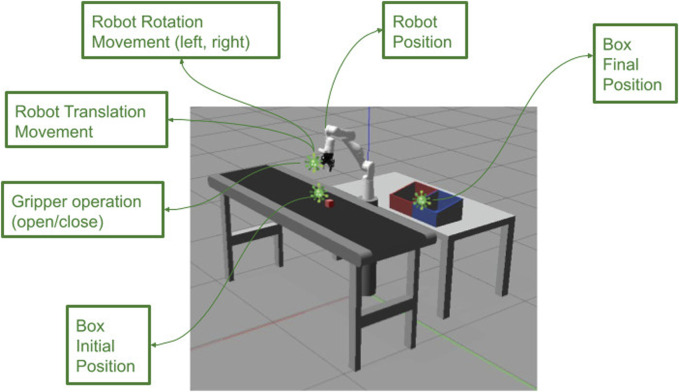
Localization and operation that we applied to the mutation.

Our approach for assessing our methodology involved following the guidelines specified in Table 1. It is worth mentioning that we customized these to align with the language and instrumentation utilized in robotics and simulation. Our evaluation entailed generating mutants that pertained to rotation (orientation modifications) and translation (position adjustments) operations for the robot, as well as variations in the initial and final positions for both the robot and the box. Furthermore, we created mutants for the gripper operations, comprising opening and closing actions (Figure 4).

Table 2 shows the 26 mutants created using our guideline and the adaptation needs for the specific robot used in our experiment.

**TABLE 2:** List of mutants.

Mutant number	Number	Description
#1	Translation	Change the y-value in translation
#2	Rotation	Change the angle orientation in rotation
#3	Translation	Change the z-value in translation
#4	Gripper operation	Do not change the gripper status
#5	Gripper operation	Change the gripper status twice
#6	Gripper operation	Do not change the gripper status with the opposite expected operation
#7	Rotation	Change the angle orientation in rotation
#8	Translation	Change the x-value in translation
#9	Robot initial position	Sensor reading with the opposite expected value for the x-component
#10	Robot initial position	Sensor reading with the opposite expected value for the y-component
#11	Robot initial position	Sensor reading with the opposite expected value for the z-component
#12	Robot initial position	Sensor reading with noise in the x-component
#13	Robot initial position	Sensor reading with noise in the y-component
#14	Robot initial position	Sensor reading with noise in the z-component
#15	Box initial position	Sensor reading with the opposite expected value for the x-component
#16	Box initial position	Sensor reading with the opposite expected value for the y-component
#17	Box initial position	Sensor reading with the opposite expected value for the z-component
#18	Box initial position	Sensor reading with noise in the x-component
#19	Box initial position	Sensor reading with noise in the y-component
#20	Box initial position	Sensor reading with noise in the z-component
#21	Box initial position	Sensor reading with the opposite expected value for the x-component
#22	Box initial position	Sensor reading with the opposite expected value for y-component
#23	Box initial position	Sensor reading with the opposite expected value for the x-component
#24	Box initial position	Sensor reading with noise in the x-component
#25	Box initial position	Sensor reading with noise in the y-component
#26	Box initial position	Sensor reading with noise in the z-component

**FIGURE 5:**
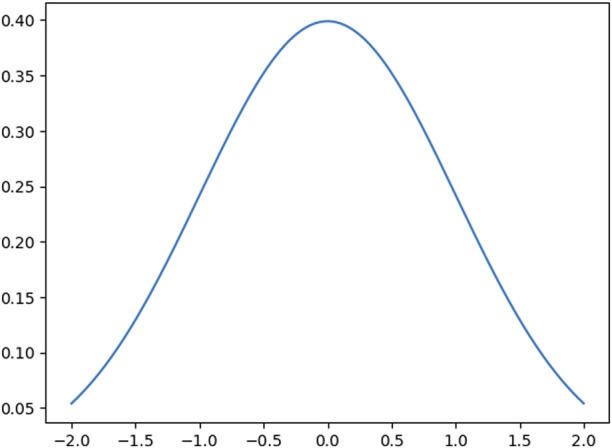
Noise added to the sensor reading.

For the noise added to sensor readings, we added the noise shown in Figure 5. This noise is a simulation of a Gaussian noise normally distributed, often used to model measurement errors or communication noise. As we can see, the lowest value for this sign is around *0.053* and the biggest is around *0.39*.

In order to guarantee the randomness of the noise added and diversity of the boxes, we conducted the test suite for each of the 26 mutants five times. The mutant scores for each round are presented in Table 3.

**TABLE 3:** Mutant score for each round.

Round	MutantScore
#1	81%
#2	77%
#3	81%
#4	81%
#5	77%

## 6 Discussion

During our experiment, the industrial robot (IR) was tasked with picking boxes from a conveyor and delivering them to designated points. To thoroughly evaluate the effectiveness of our methodology, we conducted a test suite that included the original code and 26 carefully selected mutants, representing a wide range of potential solutions. This comprehensive evaluation provided valuable insights into the efficacy of our approach.

We obtained an average score of **79%** effectiveness. Analyzing the surviving mutants in all 130 executions, those that survived were **#5**, **#10**, **#14**, **#20**, and **#24** in different rounds.

The fifth mutation affects the gripper’s operation, causing it to close twice instead of once. This change persisted as it did not interfere with the robot’s mission. However, if time is a crucial factor in meeting our acceptance criteria, the additional operation may affect the overall mission execution time.

A reversal in the error value for the y-component is the transition for **Mutant #10**. Although the expected value stands at 0.002 cm, the acceptance criteria (AC) specifies 0.02 cm. Despite this, the sensor reading remained within the acceptable threshold due to the introduced error. This highlights the **importance of aligning the definition of acceptance criteria in the business requirement (BR) with the magnitude order of variables utilized in the process**. Such alignment directly influences the resulting outcomes.

Listing 2 All the statements created at feature are contemplated by a test in the test file.

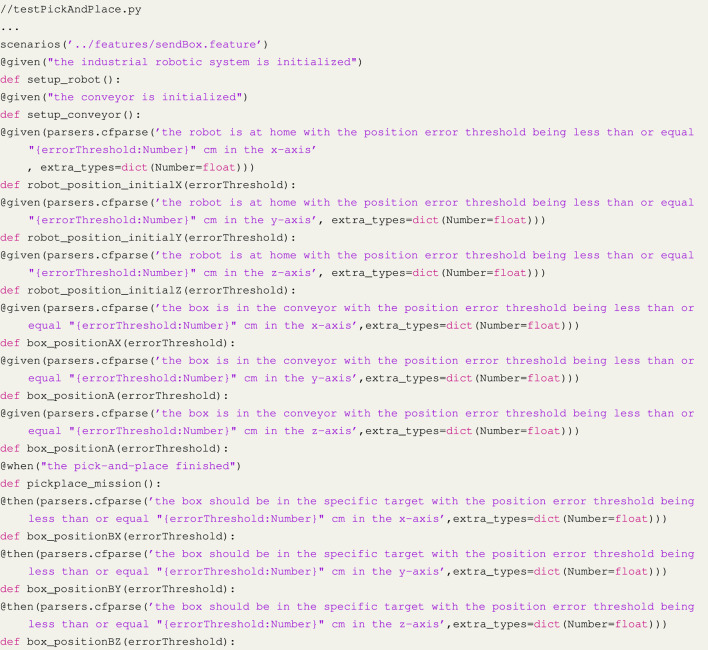

Listing 3 An example for the test using the AC.

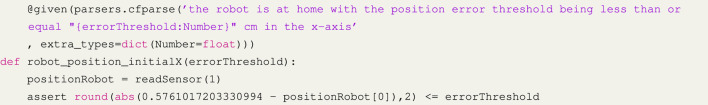

We utilized a randomized sorting method to introduce variability into the noise added to sensor readings. However, we discovered that for surviving mutants **#14**, **#20**, and **#24**, the noise added was within the established error threshold of the acceptance criteria. Consequently, we determined that aligning the acceptance criteria in the BR with the physical attributes of the instrumentation is crucial. This alignment directly impacts the outcomes. Thus, when defining the acceptance criteria in our experiment, it is imperative to consider the accuracy of the sensors.

Our analysis also revealed that adopting a natural language approach to define AC in our methodology can provide significant benefits for business analysts, developers, and testers throughout the system development lifecycle since they are also observed when application acceptance testing from BDD is applied in conventional systems. This approach generates a report through the when application acceptance testing process that serves as living documentation, accessible to the entire team. Importantly, this living documentation is not just a snapshot but is consistently updated to reflect the latest version of the application, making it an invaluable resource for the team. As highlighted by Afzal et al. (2020), the complexity of designing and crafting tests for software systems requires effective channels for coordination, collaboration, and documentation within robotic systems development teams. Adopting AAT is an excellent strategy for addressing these challenges. However, it needs qualitative studies with different stakeholders.

## 7 Threats to validity

Validity threats usually occur in a mapping study, and it was no different in our study. We highlight some of these threats and the mechanism we applied to address them.

*Mutant generation.* We created mutants with just one transition for each mutant. This was acceptable because of the nature of the acceptance test for each scenario. The tests implementing each sentence used in our approach (Given–Then–When) are executed in sequence. Thus, when one test fails, the process is stopped—if the test that implements the *Given* sentence fails, the process stops, and the test report is generated. Furthermore, no more tests are executed. Therefore, the unique transition for each mutant was an acceptable method for creating mutants to evaluate our approach.

*Input data.* As observed in the experiment, sensor characteristics were the reason for the survived mutant. Our approach is not concerned with the input data, but we strongly suggest that domain experts must also choose the input data. Moreover, our experiment performed a well-known process in the robotic field, and we used near-accurate data. Thus, further experiments with more realistic input data will be necessary to confirm the effectiveness of our approach.

## 8 Conclusion

The present research outlines an approach to automated acceptance testing (AAT) that aims to improve fault detection in industrial robotic systems (IRS). However, one challenge to applying software testing for robotic systems is related to communication and collaboration: the culture of testing.

Our study utilized an approach based on behavior-driven development (BDD); more specifically, AAT that uses natural language. Our implementation used ROS, Gazebo, and pytest-bdd, a Python library dedicated to BDD. To evaluate the effectiveness of our software testing approach, we tested the generated test suites against mutants created from the original code. The test suites produced using AAT4IRS achieved an effectiveness score of 79%.

In our assessment, we utilized mutation testing to generate mutants that accurately reflect the complexities of the robotic landscape. Our thorough methodology entailed creating mutants that focused on non-deterministic elements that are inherently present in robotic systems, such as fluctuations in sensor readings, as well as mutants that accounted for linguistic subtleties. By employing this nuanced approach, we were able to gain valuable insights into the robustness and flexibility of our proposed methodology within the constantly evolving field of robotics.

When evaluating business requirements for industrial robotic systems, it is crucial to consider both the robot’s physical attributes and overall business objectives. Achieving alignment across all teams involved in the project, which may include individuals from various backgrounds, is essential when establishing acceptance criteria (AC). Additionally, utilizing live documentation made possible by AAT4IRS implementation can help foster collaboration among teams, allowing for more effective problem-solving when facing the complex challenges of these types of projects.

Our aim for future research is (i) to apply our approach by performing controlled experiments with a group of roboticists, (ii) to apply and evaluate AAT4IRS using physical IRS, (iii) and to perform a qualitative evaluation with different stakeholders.

## Data Availability

The original contributions presented in the study are publicly available. This data can be found here: https://github.com/mgdossantos/aat4irs_v3.
